# Left External Iliac Vein Injury During Laparoscopic Pelvic Lymphadenectomy for Early-Stage Ovarian Cancer: Our Experience and Review of Literature

**DOI:** 10.3389/fsurg.2022.843641

**Published:** 2022-03-09

**Authors:** Raffaele Tinelli, Miriam Dellino, Luigi Nappi, Felice Sorrentino, Maurizio Nicola D'Alterio, Stefano Angioni, Giorgio Bogani, Salvatore Pisconti, Stefano Uccella, Erica Silvestris

**Affiliations:** ^1^Department of Obstetrics and Gynecology, “Valle d'Itria” Hospital, Martina Franca, Taranto, Italy; ^2^Department of Gynecology Oncology, “Istituto di Ricovero e Cura a Carattere Scientifico (IRCCS) Giovanni Paolo II”, Bari, Italy; ^3^Department of Medical and Surgical Sciences, Institute of Obstetrics and Gynaecology, University of Foggia, Foggia, Italy; ^4^Department of Surgical Science, Cittadella Universitaria Blocco I, Asse Didattico Medicina P2, University of Cagliari, Cagliari, Italy; ^5^Department of Obstetrics and Gynecology, University Medical School “La Sapienza”, Rome, Italy; ^6^Department of Medical Oncology, National Oncology Institute “Moscati”, Taranto, Italy; ^7^Department of Obstetrics and Gynecology, Azienda Ospedaliera Universitaria Integrata Verona, Verona, Italy

**Keywords:** injury, iliac vein, laparoscopy, lymphadenectomy, repair, ovarian cancer

## Abstract

Laparoscopic surgical staging is the standard treatment of early-stage ovarian tumors with similar survival outcomes if compared with laparotomic procedures. In this article, we report a case regarding an incidental external iliac vein injury during a pelvic lymphadenectomy for fertility sparing treatment of early-stage ovarian cancer with a video showing the laparoscopic repair without any consequence or side effect. A 36 year-old obese woman with Body Mass Index 30 kg/m^2^ referred at our hospital with an histological diagnosis of high grade ovarian serous carcinoma after a left laparoscopic salpingo-oophorectomy performed in another hospital. After an hysteroscopy with endometrial biopsy, a laparoscopic surgical staging with a pelvic and aortic lymphadenectomy with lymph-node dissection until the left renal vein, omentectomy, and appendectomy were performed. A thermal injury to the left external iliac vein occurred using the bipolar forceps during lymphadenectomy and was repaired after an immediate clamping of the site using endoclinch and the suction irrigator probe. The laceration on the iliac vein was successfully repaired using 10 mm laparoscopic titanium clips; after a follow-up of 42 months no recurrence was detected. In conclusion, laparoscopy is a safe and effective therapeutic option for fertility sparing treatment patients with early stage ovarian carcinoma with a significantly low morbidity and postoperative hospitalization, but it should be reserved for oncologic surgeons trained in advanced laparoscopic procedures and repair of vascular injuries potentially associated with high mortality rate.

## Introduction

Vascular interventions are frequently performed during gynecological oncological procedures for injury of pelvic blood vessels. Vascular repairs during gynecologic procedures are associated with an high morbidity and mortality and are associated to intraoperative vascular injury (1, 2).

Pelvic and aortic lymphadenectomy have a prognostic and therapeutic significance and are often performed during gynecological oncological procedures (1–3) operating in proximity to multiple vascular structures.

In a recent prospective study Uccella et al. (2) assessed safety and feasibility of the sentinel lymph node (SLN) technique with indocyanine green to identify the presence of lymph node metastases in patients with early stage epithelial ovarian cancer, although they observed that the detection of SLN in early epithelial ovarian cancer is low when patients are submitted to delayed-staging surgery. However, they concluded that SLN procedure is safe and feasible and has the potential to provide reliable and useful information on lymph nodal status in the majority of patients with a lower morbidity and hospitalization.

Given the high mortality associated with vascular repairs, strategies for increasing the availability of gynecological surgeons trained in vascular surgery should be adopted (2).

These complications mostly involve iliac veins (2, 3) for their anatomical variability and a deep positions with risks of serious intraoperative hemorrhage of about 2.21–4.44% (4–6).

In this article, we report a case regarding an incidental external iliac vein injury during a pelvic lymphadenectomy for fertility sparing treatment of early-stage ovarian cancer with a video showing the laparoscopic repair performed without any consequence or side effect.

## Materials and Methods

A 36 year-old obese woman with Body Mass Index 30 kg/m^2^ referred at our hospital with an histological diagnosis of high grade ovarian serous carcinoma after a left laparoscopic salpingo-oophorectomy performed in another hospital.

After an hysteroscopy with endometrial biopsy, a laparoscopic surgical staging with pelvic and aortic lymph-node dissection until the left renal vein, omentectomy and appendectomy was performed.

The pelvic lymphadenectomy started developing the right paravesical and pararectal spaces. The round ligament was coagulated and transected and the peritoneal layers of the broad ligament were opened.

Dissection of the paravesical space was completed by introducing the endoclinch and the bipolar forceps (BiClamp, 210 ERBE VIO System, Tübingen, Germany) in the space laterally to the internal iliac artery (obliterated umbilical artery) and external and common iliac lymph nodes were removed.

The obturator nerve and vessels were isolated and skeletonized and, finally, superficial, and deep obturator lymph nodes were dissected and removed.

**Figure 1:**
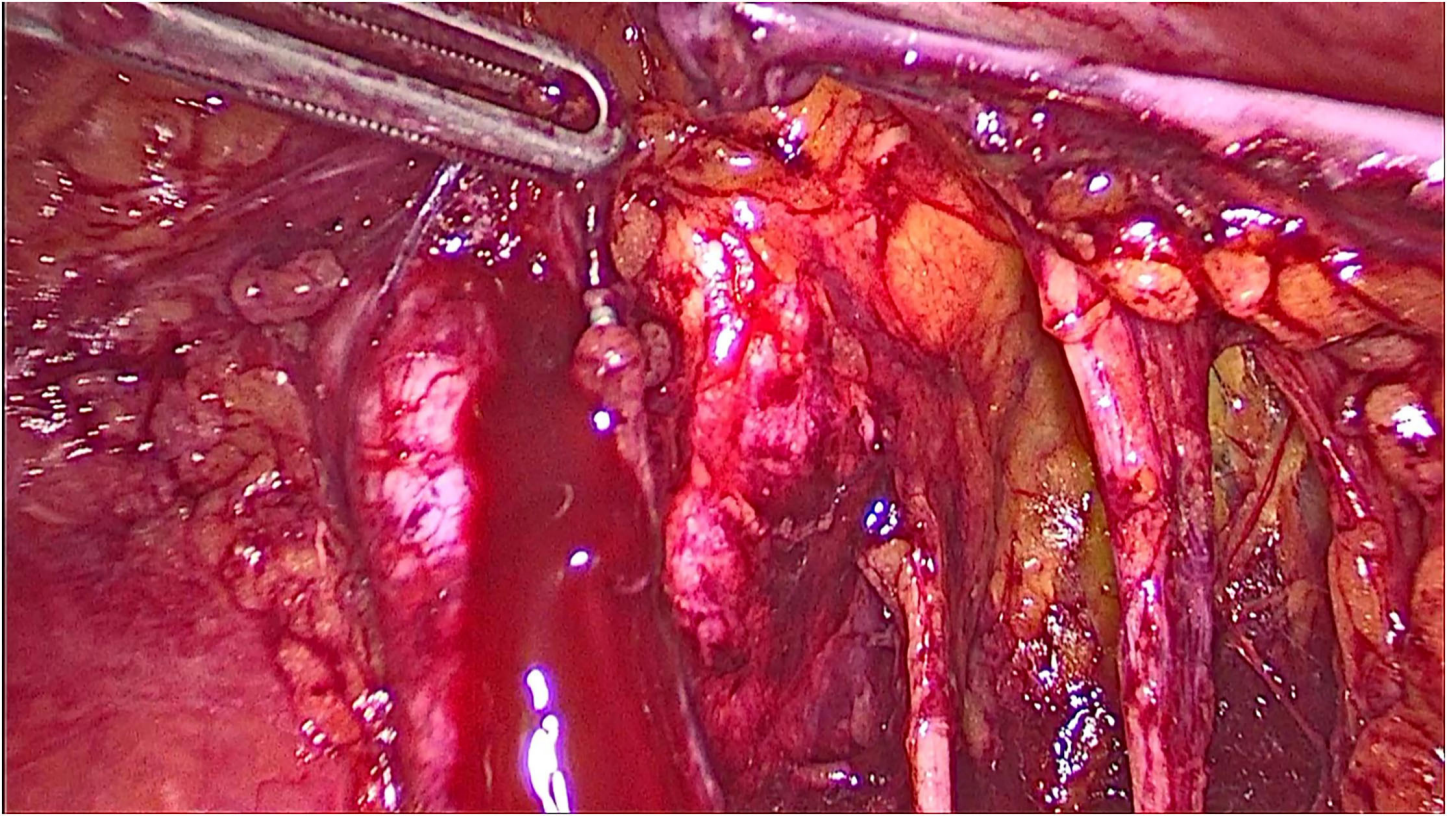
A thermal injury at the medial surface of the left external iliac vein occurred while using the bipolar forceps during left pelvic lymphadenectomy.

During the left pelvic lymphadenectomy, a thermal injury at the medial surface of the left external iliac vein occurred using the bipolar forceps (Figure 1).

After an immediate clamping of the injury site using the endoclinch and the suction irrigator probe, the insufflation pressure was increased to 20 mm Hg to reduce bleeding (1, 2). After obtaining a better view by insinuating the suction irrigator probe and drawing the blood, we decided to try a laparoscopic management of the complication.

All the procedures for unexpected surgical vascular injury were activated: after releasing the tension of the Endoclinch the entity and the site of vascular lesion were recognized and detected.

The laceration on the left external iliac vein was successfully repaired using 10 mm laparoscopic titanium clips and the operative time for the left external iliac vein repair was 3 min.

Laparoscopic vessel repair was performed successfully without intracorporeal suture; to prevent thromboembolism, intravenous heparin was administered during the surgical procedure.

The patient was regularly discharged 4 days after laparoscopic procedure without blood transfusion with an hemoglobin value that was 11 g/dL.

Low molecular–weight heparin 4,000 U subcutaneously once daily was administered during hospitalization. No sign of thromboembolism was detected; no metastases were detected by intraoperative and definitive pathologic examination; after a follow-up of 42 months no recurrence was detected.

## Discussion

Vascular injuries can occur at entry during gynecological oncological procedures using a Veress needle or at the introduction of trocars and surgeons may suspect these damages when there is any evidence of bleeding or hematoma that cannot be explained (7).

External iliac vessel injury at the beginning of a laparoscopy is frequently due to the position of the aorta and vena cava near the umbilicus; frequently, this risk is highest in thinner patients due to the proximity of the vessels to the skin (1, 2).

Although data from randomized controlled trials has shown that laparoscopic hysterectomy results in reduced operative morbidity, shorter hospital stays, and similar oncologic outcomes when compared with laparotomy, this approach may be unsuccessful in patients with obesity because of technical challenges, limited anatomical exposure due to fat deposits around iliac vessels during lymphadenectomy, and cardiopulmonary compromise while in the Trendelenburg position (3, 4).

Vascular damages in gynecology are rare (0.2/1,000) but are associated with a 6–13% of mortality.

The most frequent sites of hemorrhage are iliac vessels, inferior vena cava, and abdominal aorta.

Frequently, electrosurgery during laparoscopy may cause vascular trauma and thermal injuries: in fact injury to iliac vessels due to thermal spread are frequently reported during laparoscopic and robotic lymph node dissection and are due to failure of the insulation around the scissors (8).

Managing of these complications includes immediate recognition and localization of the injury because vascular injuries are rare but can be fatal and may require midline laparotomy and vascular surgeons (9).

The vascular iatrogenic repairs during extensive oncologic laparoscopic procedures are increasing in frequency (5–7).

Clinical evaluation of severity of the injury should be immediately done: in cases of damage to the aorta, iliac vessels or IVC (inferior vena cava), a laparotomic repair should be performed.

Injuries to aortic branch like inferior mesenteric artery that is clearly visualized may be repaired laparoscopically. However, in case of aortic branch vessel damage, skeletonization could be difficult and there is a risk of concomitant visceral injury.

Control of the bleeding by applying a direct pressure on the injury site should be considered and it will nearly always suffice.

If the site of hemorrhage cannot be visualized, abdominal packing should be done: in this case a conversion from laparoscopic to open surgery with a midline laparotomy should be considered.

Most vascular injuries will occur in the pelvis and, after pack removal out of the pelvis for definitive management, it is important to determine the extent of injury.

Common iliac artery or distal aorta are best repaired with primary suture by a vascular surgeon. These injury sites should be compressed until a vascular surgeon is available.

Repair of internal iliac artery can be performed by sutures and ligaclip because there is a great collateral blood vascularization.

Venous pelvic injuries are the most difficult to manage: in the first instance the bleeding site should be carefully packed and, after a temporarily closing of the abdomen, the patient should be re-operated the next day for pack removal and re-assessment.

If packing alone is not enough and the damage includes the sacral venous plexus, internal iliac vein or collateral veins then hemorrhage occluder pins can be directly applied to the hemorrhage site (8–10).

In a recent report Levin et al. investigated the incidence of vascular injury in patients who underwent open, laparoscopic and vaginal gynecologic surgery.

In their multicenter study including 201,224 surgical procedures they observed that vascular repairs were rare and occurred during laparotomic hysterectomies performed for malignant in patients with chronic anticoagulation, ASA class 3 or class 4 (0.2% of open, 0.04% of minimally invasive, and 0.03% of vaginal operations) (11).

In another multicenter study, of 482 gynecologic laparoscopies for benign or malignant disease performed in a USA center between 1996 and 2000, 1,165 laparoscopic hysterectomies for benign disease in Finland between 1993 and 1994, and 25,764 gynecologic laparoscopies for benign or malignant disease in the Netherlands, a 1.5, 1.2, and 0.25% of vascular injury was observed, respectively (10, 11).

A recent systematic review by King et al. on 1,097 patients reported the rate of vascular injuries during gynecologic laparoscopic procedures for benign disease. One hundred and seventy-nine vascular injuries were reported with a percentage of 0.09%. The inferior epigastric vessels were the most commonly injured and the majority of injuries occurred during abdominal entry. Most injuries were recognized intraoperatively. Only two of the 179 major vascular injuries resulted in death with a mortality rate of 0.001%.

They concluded that the incidence of major vascular injury during gynecologic laparoscopy is very low, and that laparoscopy is a safe surgical technique if performed for benign gynecologic disease (11–13).

In recent studies regarding patients who underwent laparoscopic gynecologic surgery a correlation between lower BMI and increased injuries rate was observed in thinner patients.

In fact, in patients with lower BMI a shorter distance between the skin and retroperitoneal vessels was observed as well as an increased proximity of the aortic bifurcation to the level of the umbilicus that minimize the safe distance with an higher risk of vascular injury at introduction of the Verses needle and the trocar.

Long-term multicenter studies analyzing the association between vascular injury and morbidity or mortality in gynecologic patients are not available (12, 13).

The feasibility, safety, and efficacy of laparoscopic treatment of gynecologic malignancies has been reported by several articles in the last decades (3, 4) with a lower postoperative morbidity.

In fact, staging procedures including hysterectomy, pelvic and Para aortic lymph-node dissection, omentectomy, and peritoneal biopsy are actually completed by laparoscopy (14, 15).

In our study, the hemorrhage was controlled with endoscopic clips applied distally and proximally to the lesion, with the clip applier/remover (Aesculap, Braun, Germany) introduced into the abdomen through the surgeon trocar.

In fact, endoscopic clips are safe and effective therapeutic tools that ensure a great control of hemorrhage.

Laparoscopic treatment of vascular injuries is an effective and safe procedure: to better control the hemorrhage we suggest using immediately, in case of vascular injury, the endoclinch forceps applied directly to the lesion and to isolate the vessel that have to be repaired (16–18).

In selected cases before removing the endoclinch forceps, a laparoscopic bulldog clamp can be placed proximally, and another clamp should be used distally for a better repair and an improved suture.

During last decades several articles confirmed the role of laparoscopic and robotic pelvic lymphadenectomy in the treatment of gynecologic cancers (18, 19).

Current guidelines for complete surgical staging in early-stage ovarian cancer recommend systematic lumbo-aortic and pelvic lymphadenectomy, despite their controversial therapeutic value. Sentinel lymph node (SLN) detection with indocyanine green in early ovarian cancer is a feasible technique and could provide useful information on nodal status, avoiding future lymphadenectomy in the majority of patients. SLN biopsy in ovarian cancer is not yet the standard approach, and is currently under investigation. A recent report by Turco et al. confirmed the feasibility of laparotomy approach for SLN detection in voluminous ovarian cancer (6), although a recent prospective study by Uccella et al. observed that the detection of SLN in early epithelial ovarian cancer is low when patients are submitted to delayed-staging surgery but SLN procedure could avoid systematic lymphadenectomy in the majority of patients (2, 6).

However, strategies for increasing the availability of gynecological surgeons trained in vascular surgery should be adopted (4, 5, 20, 21).

In conclusion, laparoscopy is a safe and effective procedure for treatment of fertility sparing treatment patients with early stage ovarian carcinoma with a lower morbidity and hospital stay and a reported pelvic vessel injury rate during pelvic lymphadenectomy of about 1%.

Repair of this complications should be reserved for gynecologic surgeons trained in advanced laparoscopic procedures and repair of vascular damages potentially associated with an high mortality rate.

Strategies for prevention and treatment, including continuous training on this gynecological emergency situation should be adopted to increase the availability of gynecologic surgeons trained in vascular surgery.

## Data Availability Statement

The original contributions presented in the study are included in the article/Supplementary Material, further inquiries can be directed to the corresponding author/s.

## Ethics Statement

Written informed consent was obtained from the individual(s) for the publication of any potentially identifiable images or data included in this article.

## Author Contributions

RT, LN, MiD, ES, and FS were a major contributor in writing the manuscript. SA, MaD, SP, SU, and GB were in charge of the final approval of the version to be published. RT performed the surgery. All authors analyzed, interpreted the patient data according to the histological examination and the literature review, and read and approved the final manuscript.

## Conflict of Interest

The authors declare that the research was conducted in the absence of any commercial or financial relationships that could be construed as a potential conflict of interest.

## Publisher's Note

All claims expressed in this article are solely those of the authors and do not necessarily represent those of their affiliated organizations, or those of the publisher, the editors and the reviewers. Any product that may be evaluated in this article, or claim that may be made by its manufacturer, is not guaranteed or endorsed by the publisher.

## Supplementary Material

A video reported an incidental external iliac vein injury during a pelvic lymphadenectomy for early-stage ovarian cancer that was laparoscopically repaired without any problem.
